# Professional Education in the United States

**Published:** 1893-06

**Authors:** 


### PROFESSIONAL EDUCATION IN THE UNITED STATES.

Dr. Bayard Holmes, of Chicago, has prepared an interesting and valuable paper on the above subject based upon the "Forthcoming Report of the Bureau of Education." He is pleased to see gratifying improvement in many directions. In France and Germany there has been a great advance in medicine during the past fifteen years. As medicine has become more of a science, and especially as pathology became more carefully studied, and as surgery became, under the use of antiseptic methods, more successfully practiced, the number of aspirants for a medical career has rapidly increased.

In France and Germany the medical schools are departments of the universities. In America, where this is not the case, where the professions are open to applicants possessing only the rudiments of a "common school education," where the course of study is short, and a diploma (in medicine) entitles the holder to practice, the ratio of medical students is absolutely and relatively greater than in either of the above countries.

Our medical schools are not adequately endowed, and medicine is neglected by the universities of the United States. This is made apparent by the figures giving the amount of financial support schools of medicine receive from university corporations and from States.

The length of the medical course was hardly three years (in 1890). The course of study is further abbreviated by the absurd custom of having the schools open only half the year. This means that almost $5,000,000 lie unproductive one-half of the year. It also means that in the United States in 1890, 4,500 men were given the medical degree after studying less than eighteen months in a medical school.

"Is it any wonder, then," Dr. Holmes inquires, "that educated men who are trained in our better class of colleges and scientific schools turn away from medicine in disgust?" A relatively smaller number of medical students have (in 1890) the bachelor's degree than in 1880, though the education of the average medical student is superior to that of the student ten years ago.

Medicine, therefore, does not seem to be attractive to the college-bred man. That this is not inherently the case appears from the fact that in Germany medicine is the most popular profession in the opinion of the graduates of the universities. The fault then must be with the medical schools themselves. Antique methods still prevail in the majority of medical schools to-day. The courses of lectures are so arranged that each student hears every lecture repeated the second year of his attendance and then passes his examination and graduates. The schools are too often run for the good and profit, if not for the glory, of the professors.

